# Substance Use Outcomes from Two Formats of a Cognitive-Behavioral Intervention for Aggressive Children: Moderating Roles of Inhibitory Control and Intervention Engagement

**DOI:** 10.3390/brainsci11070950

**Published:** 2021-07-19

**Authors:** John E. Lochman, Caroline L. Boxmeyer, Chuong Bui, Estephan Hakim, Shannon Jones, Francesca Kassing, Kristina McDonald, Nicole Powell, Lixin Qu, Thomas Dishion

**Affiliations:** 1 Department of Psychology, University of Alabama, Tuscaloosa, AL 35487, USA; eahakim@crimson.ua.edu (E.H.); fkassing@crimson.ua.edu (F.K.); klmcdonald2@ua.edu (K.M.); 2 Center for Youth Development and Intervention, University of Alabama, Tuscaloosa, AL 35487, USA; jones178@ua.edu (S.J.); npowell@ua.edu (N.P.); lxqu@ua.edu (L.Q.); 3 Alabama Life Research Institute, University of Alabama, Tuscaloosa, AL 35487, USA; cnbui@ua.edu; 4 Department of Psychiatry and Behavioral Medicine, University of Alabama, Tuscaloosa, AL 35487, USA; boxmeyer@ua.edu; 5 REACH Institute, Department of Psychology, Arizona State University, Tempe, AZ 85281, USA; tomd@darkwing.uoregon.edu

**Keywords:** substance use, aggression, cognitive-behavioral, group intervention

## Abstract

Although cognitive-behavioral interventions have reduced the risk of substance use, little is known about moderating factors in children with disruptive behaviors. This study examined whether aggressive preadolescents’ inhibitory control and intervention engagement moderates the effect of group versus individual delivery on their substance use. Following screening for aggression in 4th grade, 360 children were randomly assigned to receive the Coping Power intervention in either group or individual formats. The sample was primarily African American (78%) and male (65%). Assessments were made of children’s self-reported substance use from preintervention through a six-year follow-up after intervention, parent-reported inhibitory control at preintervention, and observed behavioral engagement in the group intervention. Multilevel growth modeling found lower increases in substance use slopes for children with low inhibitory control receiving individual intervention, and for children with higher inhibitory control receiving group intervention. Children with low inhibitory control but who displayed more positive behavioral engagement in the group sessions had slower increases in their substance use than did similar children without positive engagement. Aggressive children’s level of inhibitory control can lead to tailoring of group versus individual delivery of intervention. Children’s positive behavioral engagement in group sessions is a protective factor for children with low inhibitory control.

## 1. Introduction

Youth with behavioral symptoms of the disruptive behavior disorders of conduct disorder (CD) or oppositional defiant disorder (ODD) use substances earlier than peers and are more likely than their peers to develop substance use problems [1,2], and, once established, these substance use problems can remain stable through middle adulthood [3]. In a meta-analysis examining how childhood psychiatric disorders were a risk factor for later substance abuse, Groenman and colleagues [4] found that children with CD or ODD were at increased risk of developing a drug-related disorder compared to children without these diagnoses. One common factor underlying both CD and substance use was behavioral undercontrol or dysregulation [4]. Furthermore, both child externalizing behavior and behavioral undercontrol have been found to independently predict problematic substance use in adolescence [5]. Thus, it may be particularly important to consider how interventions designed to prevent CD conduct disorder are useful in preventing or prolonging onset to substance use initiation in adolescence, and how behavioral control processes may work in conjunction with interventions.

### 1.1. Prevention and Treatment of Substance Use in Aggressive Youth

Preventive and treatment interventions with aggressive and conduct problem youth can affect their substance use behaviors. When the effects of outpatient treatment on youth who have already established substance abuse have been examined through meta-analysis, group counseling has been found to be effective in reducing marijuana and mixed substance use [6]. Multidimensional and family systems interventions have also demonstrated a positive impact on substance use in youth with conduct problems and delinquent behavior. In a meta-analysis, Baldwin, Christian, Berkeljon, and Shadish [7] found that family therapy interventions for adolescents with behavioral and substance use problems were more effective than no-treatment control and were modestly more effective than care-as-usual and other forms of treatment. As an example of one such comprehensive family- and community-based treatment for youth with serious conduct problems, multisystemic therapy (MST) has been found to reduce substance use in adolescents with conduct problems and delinquent behavior [8,9,10] and in adolescents who are involved in juvenile drug court services [11].

With regard to preventive interventions, several interventions have been shown to reduce substance abuse in at-risk aggressive youth. Godwin and the Conduct Problems Prevention Research Group [12] found that Fast Track (FT), a comprehensive long-lasting program designed to decrease aggression and delinquency with at-risk kindergartners who were then followed in preventive intervention through 10th grade, decreased the probability of hazardous drinking in adolescence and young adulthood as well as opioid use in young adulthood. FT intervention-driven improvements in children’s interpersonal, intrapersonal, and academic skills were found to partially mediate the program’s direct effects on adolescent and young adult outcomes, with a strong indirect pathway through children’s earlier acquisition of interpersonal skills. Reviews of research on briefer, school-based prevention programs addressing youth’s social, coping, and social resistance skills have also found that prevention programs led to reduced risk for drug and alcohol problems in either universal classroom programs or in groups with other at-risk youth [13,14]. Key transition points, such as transition to middle school, can be especially important opportunities for focal prevention programs to produce beneficial effects among high-risk children and families [15,16], in part because the acquired problem-solving skills can help the children to better manage frequent conflicts in middle school settings that have high rates of peer antisocial behavior and less intense teacher management [17]. One such cognitive-behavioral prevention program at the middle school transition that has demonstrated preventive effects on youth substance abuse for at-risk aggressive youth is Coping Power [18].

#### 1.1.1. Coping Power

Coping Power evolved from the Anger Coping program, a cognitive behavioral skills-training program for children that can be delivered in schools [19]. Fourth through 6th grade boys with aggressive and disruptive behavior who participated in the Anger Coping program had lower rates of drug and alcohol involvement compared to untreated peers three years postintervention, as well as higher levels of self-esteem and social problem-solving skills [20]. Coping Power (CP) is a multicomponent child [21] and parent [22] program that builds upon the Anger Coping program. In a study of 183 preadolescent boys with aggressive behavior, Coping Power produced lower rates of substance use and delinquent behavior compared to the control condition at one-year follow-up, with the strongest effects for boys who received the combined child and parent Coping Power program [18]. In an effectiveness study with 245 students with aggressive behavior in the 4th through 6th grades, children who participated in Coping Power or Coping Power plus an enhanced universal prevention component displayed reduced rates of substance use compared to children in a care-as-usual control condition [23]. After one year, children in both Coping Power conditions exhibited reductions in delinquency and substance use compared to children in the care-as-usual condition [24]. 

Coping Power intervention effects on multiple indices of problem behavior have also been found with youth diagnosed with disruptive behavior disorders [25,26,27,28,29], with students with behavior problems in dissemination and implementation studies in schools and community settings [30,31,32,33,34,35], and with children in other cultural environments [36,37,38]. When Coping Power has been implemented as a treatment for children with disruptive behavior disorders, it may also have a subsequent preventive effect for adolescent substance abuse and delinquent behavior. Zonnevylle-Bender, Matthys, Van De Wiel, and Lochman [39] found that Coping Power, when implemented with children with disruptive behavior disorder diagnoses in psychiatric outpatient clinics, reduced cigarette smoking and marijuana use initiation relative to care-as-usual five years after the start of treatment. Furthermore, Coping Power participants’ substance use was in the range of the matched healthy control group at long-term follow-up.

#### 1.1.2. Moderators of Coping Power Group Intervention for Aggressive Children 

Children’s association with peers who are engaged in antisocial behavior in adolescence becomes one of the strongest proximal risk predictors for growth in subsequent delinquency [40] and substance use [41]. These findings have raised questions about the potential ineffectiveness of group interventions that aggregate antisocial youth. In one notable example, a follow-up of the Adolescent Transitions Program found that youth randomized to a cognitive-behavioral group focusing on self-regulation resulted in improvements in observed family interaction, but, unfortunately, also resulted in increases in youth-reported smoking and teacher-reported problem behavior at school at one-year [42] and three-year follow-ups [43]. Analyses of the group conditions revealed that subtle dynamics of deviancy training during unstructured transitions in the groups predicted growth in self-reported smoking and teacher ratings of delinquency [44]. 

To examine whether and the extent to which aggregating aggressive youth in group treatment might impact treatment effects, Lochman and colleagues [45] randomly assigned aggressive youth to group or individual formats of Coping Power. The existing evidence base for group-administered Coping Power and the program’s structured cognitive-behavioral approach provided a unique and unprecedented opportunity to rigorously compare the effects of group versus individual formats. Although there were no overall iatrogenic program effects in the prior Coping Power studies, and in fact there were significant prevention effects, the group format may have minimized the strength of the intervention’s potential effects, especially for children with certain characteristics [46]. Research examining this issue, comparing group versus individual delivery formats for Coping Power, has indeed found weaker group effects for children with certain characteristics [47,48], including children’s preintervention levels of inhibitory control [45], and their behavioral engagement in group sessions [49]. The following sections focus on these two central intervention moderators: children’s inhibitory control, associated with their executive functioning, and behavioral engagement in the intervention.

##### Child Inhibitory Control 

Inhibitory control can be defined as a process of effortful or willful control of behavior, capable of regulating both approach and avoidance [50,51]. It is a central component of executive functioning and involves the active inhibition of a dominant implicit or explicit response or impulse [52]. While conceptually similar to behavioral impulsivity, inhibitory control focuses on the more positive influences of constraint and not the reckless and daring behaviors affiliated with impulsivity [53]. Thus, while the ability to inhibit behavior is necessary to respond adaptively, problems with inhibitory control have been shown to predict adolescents externalizing problems such as aggression [54] and substance use [55,56,57]. 

Inhibitory control dysfunction has been noted as a common characteristic of aggressive youth [58,59]. Youth and adolescent studies have also shown that inhibitory control may moderate the relationship between various negative emotions and externalizing problems [60,61], including substance use [62]. Specifically, in discriminating between three separate components of negative emotionality (fear, anger, depressed mood) in a sample of aggressive youth, Pardini and colleagues [62] observed that, at high levels of inhibitory control, alcohol use initiation was significantly predicted by increased levels of depressed mood. However, at moderate/low levels of inhibitory control, alcohol initiation was predicted by increased levels of anger and decreased levels of fear, suggesting the role that greater inhibitory control plays in protecting youth high in anger and low in fear from alcohol use initiation [62].

Inhibitory control has been found to moderate the effects of individual versus group delivery of Coping Power on forms of externalizing behavior [45]. Youth who were lower in inhibitory control and who received Coping Power individually showed steeper declines in teacher-rated externalizing behaviors than children low on inhibitory control in the group condition. Children high in inhibitory control benefited similarly from either group or individual Coping Power. However, this pattern was no longer significant in a four-year follow-up [46].

##### Child Intervention Engagement 

A series of studies have examined the role of children’s engagement and therapeutic alliance on Coping Power outcomes. In a study of Coping Power delivered in individual sessions, Mitchell and colleagues [63] examined the role of therapeutic alliance, as rated by independent raters of video-recorded sessions. One aspect of therapeutic alliance, child–therapist bonding (in early Coping Power sessions), predicted better externalizing behavior outcomes.

Similar findings about the positive effects of early child behavioral engagement in Coping Power has also emerged for children receiving group sessions. For example, Ellis, Lindsey, Barker, Boxmeyer, and Lochman [64] found that intervention engagement fluctuated differentially across the group Coping Power intervention for children and parents. Better child engagement early in Coping Power positively influenced parent engagement midway through the intervention. This held even after accounting for contextual risk factors in the family environment, underscoring the importance of maximizing early child intervention engagement. In the same sample, Lindsey and colleagues [65] found that children’s engagement during early Coping Power sessions was significantly related to their completion of out-of-session activities (e.g., behavioral goal attainment and Coping Power homework). Early engagement in these out-of-session activities was significantly related to later in-session engagement, which in turn predicted decreased externalizing behavior postintervention. In a study of child and therapist behaviors in group Coping Power sessions that predicted children’s slopes of externalizing behavior through a one-year follow-up, children’s negative and positive behaviors in sessions were independently coded, and children’s negative behaviors, indicating weak engagement with the intervention, predicted escalating externalizing problems by the follow-up [49].

### 1.2. The Current Study

In the current study, we sought to determine whether inhibitory control moderates the effects of intervention format (group versus individual delivery of Coping Power) on youth substance use through a long-term follow-up, six years after the completion of intervention (i.e., following the youth’s 11th grade in high school). Based on our prior findings [45], we hypothesized that children’s preintervention inhibitory control would moderate the effects of intervention format (individual versus group) on children’s long-term self-reported substance use outcomes. Children with poor inhibitory control in the group Coping Power condition were expected to have greater increases in the growth curves for substance use than children of similar risk status who received individual intervention. It was also hypothesized that, within the group format condition, children with poor inhibitory control would have slower increases in growth curves for substance use if the individual children had displayed fewer negative behaviors and more positive behaviors during the group sessions, indicating greater intervention engagement.

## 2. Method

### 2.1. Sample

Children included in the analyses were the full sample from a randomized controlled trial (RCT) examining the relative effectiveness of group and individual formats of Coping Power. The RCT involved 360 children recruited from 20 public elementary schools. Schools were matched based on demographic factors (percent receiving free or reduced-price lunch and percent minority) and within each matched pair, one school was randomly assigned to either Group Coping Power (GCP) or Individual Coping Power (ICP).

Recruitment involved screening by teachers and parents for eligibility; because teacher screenings have been found to be more predictive of later externalizing problems [66,67], they were considered the primary screening and were more stringent, whereas the parent screening was used to exclude children who showed few signs of aggression in the home setting. Fourth grade teachers completed the Reactive and Proactive Aggression Questionnaire (RPQ) [68] on each student in their classrooms. Ratings were compiled across all 20 schools, and a cutoff score corresponding to the 25th percentile was determined, indicating moderate to high levels of aggressive behavior. A randomized list of eligible children was created for each school, and families were contacted according to their placement on the list. Study procedures were described to families over the phone, and face-to-face assessments were scheduled for interested families. The Behavior Assessment System for Children, Second Edition (BASC-II) [69]. Aggression scale (parent-rated) was the second screening. Children whose parents rated them within the average range or above on the BASC Aggression scale were invited to enroll in the study. Families were contacted and assessed until six children were enrolled at each school. Of the 1131 students eligible from the teacher screening, 499 were successfully contacted. Of those, 139 were excluded because they did not schedule or missed the initial appointment (45), did not pass the parent screening (41), declined to participate (32), moved (15), were a sibling of another participant (three), or had cognitive limitations (three).

Three cohorts of 120 youth were recruited over a span of three years, resulting in a total sample of 360 students. At Time 1, the mean age for the sample was 10.17 years (range of 9.17–11.79). The race and ethnicity of the sample was as follows: 78.1% African American, 20.3% Caucasian, 1.4% Hispanic, and 0.3% other. Sixty-five percent of the sample were boys. Family income was below $15,000 for 29.9% of the sample, in the $15,000–$29,999 range for 31.8% of the sample, in the $30,000–$49,999 range for 20.5% of the sample, and above $50,000 for 17.6% of the sample.

### 2.2. Intervention

The Coping Power child component is an evidence-based manualized intervention developed by Lochman and colleagues [21] to target social-cognitive deficits in youth exhibiting aggressive behavior. Coping Power uses cognitive-behavioral strategies to address social problem solving, goal setting, emotion regulation, and social informational-processing distortions (e.g., hostile attribution bias) and challenges. The intervention consisted of 32 weekly sessions conducted at school from late spring of 4th grade into 5th grade. In the ICP condition, children met individually with a Coping Power leader for 30 min sessions. In the GCP condition, groups included the six children at each school and lasted for 50–60 min. Children in GCP also received monthly individual meetings (15–30 min each) to build rapport, assess comprehension of program materials, and address individual concerns. Both conditions covered the same content; however, specific activities were adapted to condition. For example, children in GCP practiced specific skills through roleplays with their peers and received feedback from their peers at the end of each session, while children in ICP participated in roleplays individually with the Coping Power leader and received feedback from the leader at the end of each session.

Coping Power group leaders were trained in and delivered both conditions and were provided with weekly supervision from two doctoral-level psychologists to ensure high implementation fidelity. Group leaders were also provided with monthly supervisory feedback on video recordings of their sessions to ensure treatment fidelity, quality, and consistency across conditions.

### 2.3. Procedure

Time 1 (preintervention) measures were completed during the enrollment process. Time 2 (midintervention) assessments were completed during the summer after 4th grade. Time 3 (postintervention) assessments were completed in the summer after 5th grade. Time 4 (one-year follow up), Time 5 (two-year follow up), Time 6 (four-year follow up), Time 7 (five-year follow up), and Time 8 (six-year follow-up) assessments were completed after 6th, 7th, 9th, 10th, and 11th grades, respectively. Children and parents completed interviews separately with research staff who were blind to children’s treatment condition. Parents received $50 for each assessment interview and children received $10.

### 2.4. Measures

#### 2.4.1. Inhibitory Control

Inhibitory control was assessed using the inhibitory control subscale from the parent-rated Early Adolescent Temperament Questionnaire–Parent Report (EATQ) [70] at Time 1 (preintervention). The inhibitory control subscale is an average score of eight items rated on a five-point Likert scale, with higher scores indicating greater inhibitory control. Items include waiting to start an activity when asked, waiting in line, sitting still when told to do so, refraining from smiling/laughing when inappropriate, and easily stopping an activity when asked. Internal consistency for this subscale was good in prior Coping Power samples (α = 0.78) [62].

#### 2.4.2. Substance Use

Substance use outcomes were assessed using the Center for Substance Abuse Prevention (CSAP) Study Survey, which was adapted from the California Student Survey [71]. The CSAP Student Survey is a 14-item child-report questionnaire that measures students’ attitudes toward and use of alcohol, tobacco, and other drugs. The current study focused on children’s reports of daily use of alcohol, tobacco, and other drugs. Children reported how many times they used each substance per day. Number of times used daily was summed and averaged across the three substance types. Internal consistency and test-retest reliability of youth self-reported substance use have been high with youth from 10 years of age through adolescence [72,73]. Substance use measured with the CSAP Student Survey has been found to validly relate to children’s proactive and reactive aggressive behaviors in samples of children first assessed at 10 years of age [74,75].

#### 2.4.3. Group Behavior

Behaviors in group were assessed using the Cognitive Behavioral Group Coding System (CBGCS) [76]. Trained coders used the CBGCS to make ratings of children’s behaviors from video recordings of GCP sessions. Ratings were made for each child during the first ten minutes, middle ten minutes, and last ten minutes of each session, and the ratings were aggregated for analyses. Items were rated on a five-point Likert scale. Positive child behaviors included showing involvement and interest in group discussion and activities, initiating positive and friendly interactions with other group members, and other children initiating reciprocal positive and friendly interactions toward the child. Negative child behaviors included: deviant talk about antisocial ideas or behaviors; exhibiting off-task, inattentive behavior; engaging in silly or disruptive behavior; demonstrating a negative, hostile attitude; exhibiting verbal or physical aggression; and appearing to trigger these negative behaviors in other group members. Coders were required to rate nine training videos and establish 80% agreement (agreement was defined as ratings falling within one point of the comparison rating) to establish proficiency. To ensure that agreement remained at 80% or higher for the study sessions, 7% of group sessions were double-coded. Furthermore, coders met regularly to prevent coder drift and remediation training videos were required for coders whose ratings fell below 80% agreement. Interrater reliability was adequate during coding (post-training), with agreement rates of 87.1% for child behaviors (across 146 10-min observation segments). There was acceptable internal consistency for the five-item positive behaviors variable (alpha: 0.90) and for the nine-item negative behaviors variable (0.77).

### 2.5. Analytic Strategy

Three-level growth curve models were used to examine the hypotheses. Repeated measures formed level 1, which were nested in children (level 2), nested in intervention units (i.e., three annual cohorts in each school). As each wave of data collection straddled several months, time was coded as the actual time lapse from baseline. Each adolescent, as a result, had a unique set of values for the time variable. The substance use trajectory was modeled with a quadratic time trend. The fixed intercept represented the mean baseline value. The fixed linear and quadratic time effects represented the overall trajectory. Variations in the growth parameters were partitioned into variation among children within the same intervention unit, and variation among intervention units. Models were estimated using Hierarchical Linear Modeling (HLM) 7.0 with full maximum likelihood (FML) estimation [77]. FML permitted all 360 children to be included in the growth analyses.

Regarding the hypothesized moderation effect of inhibitory control, of interest was the cross-level interaction of *IGCP* × *inhibitory control* (IGCP = 1 if individual format, and 0 if group format—the reference category) on the growth rate of substance use changes over time. The model was estimated using the full sample of 360 children. Race (1 if African American, 0 otherwise) was included as a control variable. Age and gender were nonsignificant control variables in the initial model and were dropped from the final model. For the second hypothesis regarding children’s in-session behaviors, the focus was the cross-level interactions of *inhibitory control* × *positive behavior* and *inhibitory control* × *negative behavior* on growth rates of changes in substance use over time. The model was estimated using the subsample of 180 children randomized to the group format of the intervention. Race, gender, and age were included as control variables.

## 3. Results

Table 1 provides the means and standard deviations for the youth-reported substance use outcome measure by intervention condition at each of the eight time points. To address missing data, the HLM analyses used FML to estimate model parameters, and all 360 participants were included in the analyses. The data collection rates (indicating rate of data completion at each time point, based on the percentage of the full sample at T1) were similar for GCP (99%, 94%, 86%, 78%, 79%, 76%, 78% for T2–T8, respectively), and for ICP (98%, 91%, 83%, 77%, 76%, 76%, 81% for T2–T8, respectively). There were no significant differences in the data collection rates between the two conditions at any of the seven postbaseline time points for the substance use outcome. Data collection bias was tested by examining whether children’s characteristics (gender, race, initial level of substance use at baseline) and intervention condition status differentiated those with data at each time point from those without data at that timepoint using logistic regression. There was little evidence of association between data collection rates and children’s characteristics or intervention conditions. One exception was race with African American children having higher rates of data collection at time points 4, 6, 7, and 8 than children of other races.

### 3.1. Hypothesized Moderation Effect of Inhibitory Control on ICP Versus GCP

**Figure 1:**
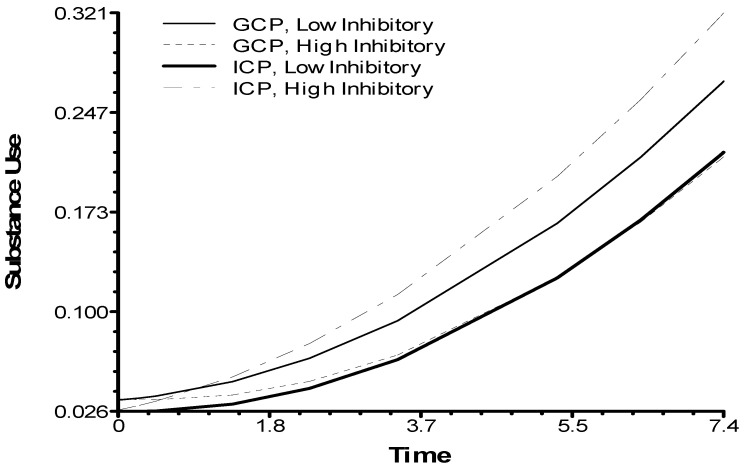
Inhibitory control moderating effects of delivery formats on youth-reported substance use.

Table 2 summarizes the results of the growth curve analysis testing the hypothesized moderation effect of inhibitory control. Race was a significant predictor of the substance use outcome, with African American youth having lower rate of increases in substance use over time than other youth. The analysis found that children’s baseline inhibitory control moderated the effect of the intervention delivery formats (*p* = 0.053). As depicted in Figure 1, youth with higher inhibitory control had lower rates of increases in substance use if they had been in the GCP condition. The reverse was true for youth with lower inhibitory control: the ICP condition had lower rates of increases in substance use compared to the GCP condition.

### 3.2. Child In-Session Behaviors Predicting Substance Use within the GCP Condition

**Figure 2:**
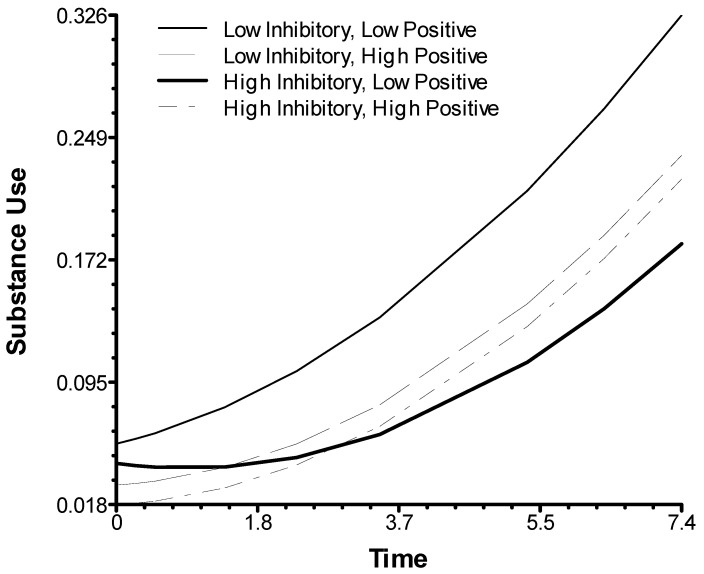
Inhibitory control moderating effects of children’s in-session behaviors on youth-reported substance use.

Table 3 summarizes the results of the growth curve analysis testing the interaction between inhibitory control and in-session positive and negative behaviors of children in the GCP condition, indicating their engagement with the intervention. The analysis found that children’s baseline inhibitory control interacted with children’s positive in-session behaviors (*p* = 0.028). As depicted in Figure 2, the combination of lower inhibitory control and lower rates of positive in-session behaviors predicted higher increases in slopes for substance use than lower inhibitory control in combination with higher rates of positive behaviors, as well as for higher inhibitory control.

## 4. Discussion

This study examined whether the format of intervention delivery (group versus individual) could influence youth substance use rates through a six-year follow-up period after a cognitive-behavioral intervention for aggressive youth had been completed and whether two hypothesized child characteristics could affect how the youth responded, in terms of substance use, to the two formats. Overall, as developmentally anticipated, slopes of youth self-reported substance use increased across time, from the intervention baseline when youth were in 4th grade through their adolescent development, ending after 11th grade. In typical adolescents, there are increases in substance use, especially from middle school to high school, similar to the findings with this current sample [78]. Although the group versus individual format of delivery did not have a significant main effect on youth self-reported substance use, children who had lower levels of inhibitory control did have slower rates of increases in their substance use if they received intervention in an individual format. Unexpectedly, the reverse pattern was evident for aggressive children with higher levels of baseline inhibitory control, as they had slower increases in their substance use slopes if they had received intervention in the group format. In addition, for children in the group format, the hypothesis predicting that risk related to low inhibitory control would be moderated by behavioral indicators of children’s engagement in intervention sessions was partially supported as children’s positive behaviors in group sessions, but not their negative behaviors, predicted a reduced rate of increase in substance use over time. Earlier research has indicated that Coping Power generally delays the onset of substance abuse in middle school [18,24], and the variability in rate of increases in substance use observed in the current study is important, as earlier first-time use of alcohol and marijuana predicts elevated substance use disorders into adulthood [78].

### 4.1. Effects of Group Versus Individual Format of Cognitive-Behavioral Intervention

Aggressive children with lower levels of inhibitory control prior to the intervention had more rapid increases in substance use through adolescence if they had received intervention in a group format rather than in an individual format. Inhibitory control is believed to be especially important in regulating the expression of negative emotions, in being able to selectively attend to key information in our environment, and in resisting temptations to act impulsively [79], all of which can contribute to children’s behavior. Children with lower levels of inhibitory control have less willful control of their behavior and anger [50] and are at risk for early increases in substance use through the adolescent years [62].

The negative association of the group format with lower levels of inhibitory control in children in this sample was evident in their rates of externalizing behavior through a one-year follow-up after intervention [45], and the current findings indicate that the group format can have a more generalized effect for these children on another key outcome, their substance use rates. Children with poor inhibitory control may be less likely to profit from being in a group intervention rather than being seen individually by the therapist for several reasons. Aggressive children with weak inhibitory control may be distracted by their peers in the group and thus be less attentive to and have resultant less recall (and internalization) of discussions and activities about key skills [45]. Children with poor inhibitory control may be more easily aroused by peers’ negative behaviors during group sessions. In addition, children with weak inhibitory control may be more easily influenced by deviancy training from antisocial peers in their group sessions [42] and less able to resist the temptation to be affected by peer reinforcement of their poorly controlled and potentially deviant talk and behavior in sessions.

Although the finding that youth with poor inhibitory control had better long-term substance use outcomes when receiving individual rather than group-based intervention was expected, the opposite pattern for children with higher levels of inhibitory control was unexpected. If children had higher levels of inhibitory control (relative to other children in this aggressive sample) they had relatively slower rates of increase in substance use if they received group intervention than if they received individual intervention. Aggressive children with greater inhibitory control may be able to better regulate their emotions and selectively attend to key group activities and tasks. Thus, they could profit more from activities that are unique to the group format, especially activities that involve coping with negative peer pressure and temptations to use substances. The Coping Power group format includes roleplay with peers on emotion regulation and problem-solving tasks and unique opportunities to receive social and tangible rewards. Children can receive rewards for the whole group if group members attain sufficient goal points, and they have potential for receiving spontaneous and structured peer social reinforcement for cooperative and positively engaged behavior in sessions. Individual delivery of Coping Power covers the same content as the group format but lacks these features that could further generalize skills and enhance motivation to try out more competent interpersonal behaviors, inside and outside the group. We had not found that an individual intervention format increased negative outcomes, in comparison to the group format, with high inhibitory control youth on other outcomes, such as externalizing behavior problems, in this sample [45]. However, substance use initiation can be encouraged by peers in important ways [2,80], and thus behavioral practice in refusing peer pressure and in positively engaging peers may be more essential for substance use outcomes.

### 4.2. Interaction of Children’s Inhibitory Control and Their Behavioral Engagement in Groups

If aggressive children have lower inhibitory control and have received cognitive-behavioral intervention in groups, they are at risk for earlier initiation and increase of substance use. However, the current findings indicate these at-risk youth have better outcomes (i.e., lower substance use slopes) if youth display higher observed positive behavior in group sessions. Youth with observed positive behavior were coded as showing involvement and interest in group discussion and activities, initiating positive and friendly interaction with other group members, and receiving reciprocal positive and friendly interactions from their peers in the group.

The protective effects of better engagement in group when a youth has low inhibitory control can occur for several reasons. When children are more actively involved in group activities and discussions, they can receive more social reinforcement from group leaders and their peers and can receive more tangible rewards if the group uses a “prize box” as youth earn participation points in their group, as is the case in Coping Power. Thus, external reinforcement can assist children who have poor inhibitory control, and who by nature may be less engaged in group tasks, to be more actively involved in group tasks involving emotion regulation and problem solving. As a result of their enhanced engagement, they can cope with some of the characteristics of their low level of general inhibitory control and have better potential attention to, and recall of, group activities and discussion. They can more firmly acquire skills that can slow their increase in substance use in the years ahead.

Finally, their positive involvement in group activities can enhance their therapeutic alliance with their group leader, and a strong therapeutic alliance has been found to be predictive of improved behavior following intervention [63]. In a related way, we have found that aggressive children who have group therapists who are engaged in warm, nonirritable ways with them are more likely to display reductions in externalizing behaviors in the years following intervention [49]. Group therapists who handle difficult interpersonal provocations from their child clients by exerting inhibitory control over their expression of their own frustration and by effectively regulating their arousal can be developing stronger therapeutic alliances with the children in the group and are modeling key processes that can be instrumental for children learning to improve their own emotional regulation over time [81]. As children’s frustration tolerance and self-regulation abilities develop due to their modeling of the group leader, they may be more likely to positively engage with group activities [49].

### 4.3. Limitations and Future Directions

There are four limitations to this study that indicate the current results can be regarded with caution and lead to future research opportunities. First, the assessment of youth substance use in the present study was youth self-report. Although youth self-report of substance use has displayed at least moderate reliability and validity during the preadolescent and adolescent age periods [72,73,74,75,82], objective measures such as biochemical corroboration using urinalysis [83] and use of parent reports would strengthen the validity of the assessment of substance use and would be useful in future longitudinal research. Second, the youth and parents received stipends to participate in assessments. Although the sizes of the stipends were not deemed to be coercive by the university’s Institutional Review Board, the stipends may have influenced parents’ and youths’ desire to participate in the study and could have skewed the sample toward having more individuals with limited incomes. Third, the current study was unable to determine if the effects were directly affected by mediating factors such as therapeutic alliance and active participation in roleplaying activities, and future research could include a methodological focus on assessment and analysis of mediating factors. Fourth, there was no untreated control or comparison group in this study. The randomized design of this study was specifically focused on differential effects of group versus individual format of intervention; therefore, it cannot confirm if either format performed better than an untreated control or comparison group through the follow-up years. Although prior randomized trial research testing the Coping Power program in comparison to control conditions has found children who had completed the full Coping Power program to have lower substance use at one- and four-year follow-ups after intervention [18,24,39], future research could determine if the Coping Power child component by itself could produce long-term relatively lower rates of increases in substance use through a six-year follow-up in comparison to a control group.

### 4.4. Clinical Implications

The differential effect for high versus low inhibitory control in group versus individual format suggests that tailoring of intervention format could be important for adolescent substance use for aggressive children who display symptoms of disruptive behavior disorders. When focused on preventing substance abuse, it is important to consider personalizing intervention format (individual versus group) depending on children’s level of inhibitory control. In addition, if an aggressive child with poor inhibitory control is receiving group-based cognitive-behavioral intervention, the current results indicate the importance of stimulating and reinforcing children’s early positive engagement in group sessions. The essential attention to children’s behavioral engagement in group intervention is consistent with prior research that has found that aggressive children’s engagement through the middle set of group sessions can predict reductions in externalizing behavior problems [65]. It is important for clinical trainings to emphasize the transformative potential of bonding and therapeutic alliance when working with youth exhibiting aggressive behaviors, even in the context of a manualized intervention [63]. The training of group leaders should not only emphasize skill training in a traditional sense but also focus on how group leaders can promote children’s positive behavioral engagement and practice emotional regulation themselves while engaged in group work that can be inherently stressful and frustrating at times [46,84].

## 5. Conclusions

When considering long-term substance use outcomes, aggressive children’s level of inhibitory control can lead to tailoring of group versus individual delivery of intervention, with children having weaker inhibitory benefitting more from individually-delivered intervention, but children with stronger inhibitory control benefitting more from group intervention. Children’s positive behavioral engagement in group sessions is a protective factor for children with low inhibitory control.

## Figures and Tables

**Table 1:** Means and standard deviations of substance use across time points.

Time	Group Format (GCP)			Individual Format (ICP)		
	Mean	S.D	*n*	Mean	S.D	*n*
1	0.05	0.26	179	0.04	0.20	180
2	0.04	0.21	179	0.03	0.13	176
3	0.02	0.11	170	0.01	0.10	163
4	0.04	0.14	155	0.05	0.26	149
5	0.11	0.34	140	0.12	0.42	139
6	0.18	0.47	142	0.20	0.62	137
7	0.17	0.54	136	0.16	0.43	137
8	0.23	0.61	141	0.29	0.66	146

**Table 2:** Growth curve analysis of the inhibitory control moderation effect on substance use.

	Fixed Effect					Random Effect								
						Level-3				Level-2				Level-1
	Coef.	SE	*t*-Value	df	*p*-Value	Var	$x^{2}$	df	*p*-Value	Var	$x^{2}$	df	*p*-Value	
Substance use														
Linear growth rate	0.001	0.011	0.06	58	0.951	0.000	50.85	58	>0.500	0.111	425.75	282	0.000	0.069
IGCP (1 = I-CP 0 = G-CP)	0.005	0.009	0.571	58	0.570									
Race (1 = African American, 0 = other)	−0.036	0.015	−2.48	175	0.014									
Inhibitory control	−0.008	0.008	−0.93	175	0.356									
IGCP × nhibitory control	0.022	0.011	1.95	175	0.053									
Quadratic curve growth rate	0.004	0.001	2.544	59	0.014									

**Table 3:** Growth curve analysis of interaction between children’s inhibitory control and in-session behaviors on rate of substance use within GCP.

	Fixed Effect					Random Effect								
						Level-3				Level-2				Level-1
	Coef.	SE	t-Value	df	*p*-Value	Var	$x^{2}$	df	*p*-Value	Var	$x^{2}$	df	*p*-Value	
Substance use														
Linear growth rate	0.003	0.014	0.19	27	0.851	0.000	26.31	27	>0.500	0.008	170.30	137	0.028	0.071
Positive behavior	0.000	0.005	0.05	27	0.964									
Negative behavior	−0.001	0.001	−0.09	27	0.930									
Race	−0.029	0.022	−1.29	80	0.202									
Age	0.004	0.011	0.34	80	0.735									
Gender	−0.009	0.014	−0.66	80	0.514									
Inhibit control	−0.007	0.009	−0.79	80	0.434									
Positive behav*inhibitory control	0.013	0.006	2.24	80	0.028									
Negative behav × inhibitory control	0.007	0.007	1.11	80	0.272									
Quadratic curve growth rate	0.003	0.002	1.722	29	0.096									

## Data Availability

Data used in this study can be obtained from the corresponding author.

## References

[1] Williams R.J., Nowatzki N.R. (2005). Validity of adolescent self report of substance use. Subst. Use Misuse.

[2] Conner B.T., Lochman J.E. (2010). Comorbid conduct disorder and substance use disorders. Clin. Psychol. Sci. Pract..

[3] Marmorstein N.R., White H.R., Lochman J.E., Matthys W. (2018). Comorbidity with substance abuse. The Wiley Handbook of Disruptive and Impulse-Control Disorders.

[4] Lansford J.E., Godwin J., McMahon R.J., Crowley M., Pettit G.S., Bates J.E., Coie J.D., Dodge K.A. (2021). Early Physical Abuse and Adult Outcomes. Pediatrics.

[5] Groenman A.P., Janssen T., Oosterlaan J. (2017). Childhood Psychiatric Disorders as Risk Factor for Subsequent Substance Abuse: A Meta-Analysis. J. Am. Acad. Child Adolesc. Psychiatry.

[6] Trucco E.M., Hicks B.M., Villafuerte S., Nigg J.T., Burmeister M., Zucker R.A. (2016). Temperament and externalizing behavior as mediators of genetic risk on adolescent substance use. J. Abnorm. Psychol..

[7] Tanner-Smith E.E., Wilson S.J., Lipsey M.W. (2013). The comparative effectiveness of outpatient treatment for adolescent substance abuse: A meta-analysis. J. Subst. Abus. Treat..

[8] Baldwin S.A., Christian S., Berkeljon A., Shadish W.R. (2011). The Effects of Family Therapies for Adolescent Delinquency and Substance Abuse: A Meta-analysis. J. Marital Fam. Ther..

[9] Henggeler S.W., Borduin C.M., Melton G.B., Mann B.J. (1991). Effects of multisystemic therapy on drug use and abuse in serious juvenile offenders: A progress report from two outcome studies. Fam. Dyn. Addict. Q..

[10] Letourneau E.J., Henggeler S.W., Borduin C.M., Schewe P.A., McCart M.R., Chapman J.E., Saldana L. (2009). Multisystemic therapy for juvenile sexual offenders: 1-year results from a randomized effectiveness trial. J. Fam. Psychol..

[11] Timmons-Mitchell J., Bender M.B., Kishna M.A., Mitchell C.C. (2006). An Independent Effectiveness Trial of Multisystemic Therapy with Juvenile Justice Youth. J. Clin. Child Adolesc. Psychol..

[12] Henggeler S.W., McCart M.R., Cunningham P.B., Chapman J.E. (2012). Enhancing the effectiveness of juvenile drug courts by integrating evidence-based practices. J. Consult. Clin. Psychol..

[13] Godwin J.W. (2020). The Fast Track intervention’s impact on behaviors of despair in adolescence and young adulthood. Proc. Natl. Acad. Sci. USA.

[14] Das J.K., Salam R.A., Arshad A., Finkelstein Y., Bhutta Z.A. (2016). Interventions for adolescent substance abuse: An overview of systematic reviews. J. Adolesc. Health.

[15] Griffin K.W., Botvin G.J. (2010). Evidence-Based interventions for preventing substance use disorders in adolescents. Child Adolesc. Psychiatr. Clin. N. Am..

[16] Botvin G.J., Baker E., Dusenbury L., Botvin E.M., Diaz T. (1995). Long-term Follow-up Results of a Randomized Drug Abuse Prevention Trial in a White Middle-Class Population. JAMA.

[17] Dishion T.J., Kavanagh K., Schneiger A., Nelson S., Kaufman N.K. (2002). Preventing Early Adolescent Substance Use: A Family-Centered Strategy for the Public Middle School. Prev. Sci..

[18] Ialongo N., Poduska J., Werthamer L., Kellam S. (2001). The Distal Impact of Two First-Grade Preventive Interventions on Conduct Problems and Disorder in Early Adolescence. J. Emot. Behav. Disord..

[19] Lochman J.E., Wells K.C. (2004). The Coping Power Program for Preadolescent Aggressive Boys and Their Parents: Outcome Effects at the 1-Year Follow-Up. J. Consult. Clin. Psychol..

[20] Larson J., Lochman J.E. (2002). Helping Schoolchildren Cope with Anger: A Cognitive-Behavioral Intervention.

[21] Lochman J.E. (1992). Cognitive-Behavioral intervention with aggressive boys: Three-year follow-up and preventive effects. J. Consult. Clin. Psychol..

[22] Lochman J.E., Wells K.C., Lenhart L.A. (2008). Coping Power: Child Group Program Workbook.

[23] Wells K.C., Lochman J.E., Lenhart L.A. (2008). Coping Power: Parent Group Program Workbook.

[24] Lochman J.E., Wells K.C. (2002). The Coping Power program at the middle-school transition: Universal and indicated prevention effects. Psychol. Addict. Behav..

[25] Lochman J.E., Wells K.C. (2003). Effectiveness of the coping power program and of classroom intervention with aggressive children: Outcomes at a 1-year follow-up. Behav. Ther..

[26] Helander M., Lochman J., Högström J., Ljótsson B., Hellner C., Enebrink P. (2018). The effect of adding Coping Power Program-Sweden to Parent Management Training-effects and moderators in a randomized controlled trial. Behav. Res. Ther..

[27] Muratori P., Milone A., Levantini V., Ruglioni L., Lambruschi F., Pisano S., Masi G., Lochman J.E. (2019). Six-year outcome for children with ODD or CD treated with the coping power program. Psychiatry Res..

[28] Muratori P., Milone A., Manfredi A., Polidori L., Ruglioni L., Lambruschi F., Masi G., Lochman J.E. (2015). Evaluation of Improvement in Externalizing Behaviors and Callous-Unemotional Traits in Children with Disruptive Behavior Disorder: A 1-Year Follow Up Clinic-Based Study. Adm. Policy Ment. Health Ment. Health Serv. Res..

[29] Nystrand C., Helander M., Enebrink P., Feldman I., Sampaio F. (2020). Adding the Coping Power Programme to parent management training: The cost-effectiveness of stacking interventions for children with disruptive behaviour disorders. Eur. Child Adolesc. Psychiatry.

[30] Pullen S.J., Horgan L., Romanelli L.H., Radin A., Gardner K., Edwards C., Crapo T., Bolen B., Huck B., Wells K. (2021). The effectiveness of training rural mental health clinicians to treat disruptive behavior disorders. J. Rural. Ment. Health.

[31] Cowell K., Horstmann S., Linebarger J., Meaker P., Aligne C.A. (2008). A “Vaccine” against violence: Coping Power. Pediatr. Rev..

[32] Eiraldi R., Mautone J.A., Khanna M.S., Power T.J., Orapallo A., Cacia J., Schwartz B.S., McCurdy B., Keiffer J., Paidipati C. (2018). Group CBT for Externalizing Disorders in Urban Schools: Effect of Training Strategy on Treatment Fidelity and Child Outcomes. Behav. Ther..

[33] Jurecska D.E., Hamilton E.B., Peterson M.A. (2011). Effectiveness of the Coping Power Program in middle-school children with disruptive behaviours and hyperactivity difficulties. Support Learn..

[34] Lochman J.E., Boxmeyer C., Powell N., Qu L., Wells K., Windle M. (2009). Dissemination of the Coping Power program: Importance of intensity of counselor training. J. Consult. Clin. Psychol..

[35] McDaniel S.C., Lochman J.E., Tomek S., Powell N., Irwin A., Kerr S. (2018). Reducing Risk for Emotional and Behavioral Disorders in Late Elementary School: A Comparison of Two Targeted Interventions. Behav. Disord..

[36] Peterson M.A., Hamilton E.B., Russell A.D. (2009). Starting well: Facilitating the middle school transition. J. Appl. Sch. Psychol..

[37] Cabiya J.J., Padilla-Cotto L., González K., Sanchez-Cestero J., Martínez-Taboas A., Sayers S. (2008). Effectiveness of a cognitive-behavioral intervention for Puerto Rican children. Rev. Interam. Psicol..

[38] Ludmer J.A., Sanches M., Propp L., Andrade B.F. (2017). Comparing the Multicomponent Coping Power Program to Individualized Parent–Child Treatment for Improving the Parenting Efficacy and Satisfaction of Parents of Children with Conduct Problems. Child Psychiatry Hum. Dev..

[39] Mushtaq A., Lochman J.E., Tariq P.N., Sabih F. (2016). Preliminary Effectiveness Study of Coping Power Program for Aggressive Children in Pakistan. Prev. Sci..

[40] Zonnevylle-Bender M.J.S., Matthys W., Van De Wiel N.M.H., Lochman J.E. (2007). Preventive Effects of Treatment of Disruptive Behavior Disorder in Middle Childhood on Substance Use and Delinquent Behavior. J. Am. Acad. Child Adolesc. Psychiatry.

[41] Tremblay R.E., Mâsse L.C., Vitaro F., Dobkin P.L. (1995). The impact of friends’ deviant behavior on early onset of deliquency: Longitudinal data from 6 to 13 years of age. Dev. Psychopathol..

[42] Price J., Drabick D.A., Ridenour T.A. (2019). Association with Deviant Peers Across Adolescence: Subtypes, Developmental Patterns, and Long-Term Outcomes. J. Clin. Child Adolesc. Psychol..

[43] Dishion T.J., Andrews D.W. (1995). Preventing escalation in problem behaviors with high-risk young adolescents: Immediate and 1-year outcomes. J. Consult. Clin. Psychol..

[44] Poulin F., Dishion T.J., Burraston B. (2001). 3-Year Iatrogenic Effects Associated with Aggregating High-Risk Adolescents in Cognitive-Behavioral Preventive Interventions. Appl. Dev. Sci..

[45] Dishion T.J., Tipsord J.M. (2011). Peer Contagion in Child and Adolescent Social and Emotional Development. Annu. Rev. Psychol..

[46] Lochman J.E., Dishion T.J., Powell N.P., Boxmeyer C.L., Qu L., Sallee M. (2015). Evidence-Based preventive intervention for preadolescent aggressive children: One-year outcomes following randomization to group versus individual delivery. J. Consult. Clin. Psychol..

[47] Lochman J.E., Glenn A.L., Powell N.P., Boxmeyer C.L., Bui C., Kassing F., Qu L., Romerro D.E., Dishion T. (2019). Group versus individual format of intervention for aggressive children: Moderators and predictors of outcomes through 4 years after intervention. Dev. Psychopathol..

[48] Glenn A.L., Lochman J.E., Dishion T., Powell N.P., Boxmeyer C., Qu L. (2018). Oxytocin Receptor Gene Variant Interacts with Intervention Delivery Format in Predicting Intervention Outcomes for Youth with Conduct Problems. Prev. Sci..

[49] Glenn A.L., Lochman J.E., Dishion T., Powell N.P., Boxmeyer C., Kassing F., Qu L., Romero D. (2018). Toward Tailored Interventions: Sympathetic and Parasympathetic Functioning Predicts Responses to an Intervention for Conduct Problems Delivered in Two Formats. Prev. Sci..

[50] Lochman J.E., Dishion T.J., Boxmeyer C.L., Powell N.P., Qu L. (2017). Variation in Response to Evidence-Based Group Preventive Intervention for Disruptive Behavior Problems: A View from 938 Coping Power Sessions. J. Abnorm. Child Psychol..

[51] Enticott P., Ogloff J., Bradshaw J.L. (2006). Associations between laboratory measures of executive inhibitory control and self-reported impulsivity. Personal. Individ. Differ..

[52] Rothbart M.K., Kohnstamm G.A., Bates J.E., Rothbart M.K. (1989). Temperament in childhood: A framework. Temperament in Childhood.

[53] Barkley R.A. (1997). Behavioral inhibition, sustained attention, and executive functions: Constructing a unifying theory of ADHD. Psychol. Bull..

[54] Colder C.R., Stice E. (1998). A Longitudinal Study of the Interactive Effects of Impulsivity and Anger on Adolescent Problem Behavior. J. Youth Adolesc..

[55] Sarkisian K., Van Hulle C., Lemery-Chalfant K., Goldsmith H. (2017). Childhood inhibitory control and adolescent impulsivity and novelty seeking as differential predictors of relational and overt aggression. J. Res. Personal..

[56] Fosco W.D., Hawk L.W., Colder C.R., Meisel S.N., Lengua L.J. (2019). The development of inhibitory control in adolescence and prospective relations with delinquency. J. Adolesc..

[57] Nigg J.T., Wong M.M., Martel M.M., Jester J.M., Puttler L.I., Glass J.M., Adams K.M., Fitzgerald H.E., Zucker R.A. (2006). Poor Response Inhibition as a Predictor of Problem Drinking and Illicit Drug Use in Adolescents at Risk for Alcoholism and Other Substance Use Disorders. J. Am. Acad. Child Adolesc. Psychiatry.

[58] Tarter R.E., Kirisci L., Mezzich A., Cornelius J.R., Pajer K., Vanyukov M., Gardner W., Blackson T., Clark D. (2003). Neurobehavioral Disinhibition in Childhood Predicts Early Age at Onset of Substance Use Disorder. Am. J. Psychiatry.

[59] Raaijmakers M.A.J., Smidts D.P., Sergeant J.A., Maassen G.H., Posthumus J.A., Van Engeland H., Matthys W. (2008). Executive Functions in Preschool Children with Aggressive Behavior: Impairments in Inhibitory Control. J. Abnorm. Child Psychol..

[60] Utendale W.T., Hastings P.D. (2011). Developmental changes in the relations between inhibitory control and externalizing problems during early childhood. Infant Child Dev..

[61] Oldehinkel A.J., Hartman C.A., Ferdinand R.F., Verhulst F.C., Ormel J. (2007). Effortful control as modifier of the association between negative emotionality and adolescents’ mental health problems. Dev. Psychopathol..

[62] Valiente C., Eisenberg N., Smith C.L., Reiser M., Fabes R.A., Losoya S., Guthrie I.K., Murphy B.C. (2003). The relations of effortful control and reactive control to children’s externalizing problems: A longitudinal assessment. J. Personal..

[63] Pardini D., Lochman J., Wells K. (2004). Negative Emotions and Alcohol Use Initiation in High-Risk Boys: The Moderating Effect of Good Inhibitory Control. J. Abnorm. Child Psychol..

[64] Mitchell Q.P., Younginer S.T., Lochman J.E., Vernberg E.M., Powell N.P., Qu L. (2020). Examining for the Protective Effects of Therapeutic Engagement on Child Aggression. J. Emot. Behav. Disord..

[65] Ellis M.L., Lindsey M.A., Barker E.D., Boxmeyer C.L., Lochman J.E. (2013). Predictors of Engagement in a School-Based Family Preventive Intervention for Youth Experiencing Behavioral Difficulties. Prev. Sci..

[66] Hogue A., Henderson C.E., Ozechowski T.J., Becker S.J., Coatsworth J.D. (2019). Can the group harm the individual? Reviewing potential iatrogenic effects of group treatment for adolescent substance use. Clin. Psychol. Sci. Pract..

[67] Hill L.G., Coie J.D., Lochman J.E., Greenberg M.T., The Conduct Problems Prevention Research Group (2004). Effectiveness of Early Screening for Externalizing Problems: Issues of Screening Accuracy and Utility. J. Consult. Clin. Psychol..

[68] Kassing F., Godwin J., Lochman J.E., Coie J.D., Conduct Problems Prevention Research Group (2018). Using Early Childhood Behavior Problems to Predict Adult Convictions. J. Abnorm. Child Psychol..

[69] Dodge K.A., Lochman J.E., Harnish J.D., Bates J.E., Pettit G.S. (1997). Reactive and proactive aggression in schoolchildren and psychiatrically impaired chronically assaultive youth. J. Abnorm. Psychol..

[70] Lindsey M.A., Romanelli M., Ellis M.L., Barker E., Boxmeyer C.L., Lochman J.E. (2019). The Influence of Treatment Engagement on Positive Outcomes in the Context of a School-Based Intervention for Students with Externalizing Behavior Problems. J. Abnorm. Child Psychol..

[71] Capaldi D.M., Rothbart M.K. (1992). Development and Validation of an Early Adolescent Temperament Measure. J. Early Adolesc..

[72] Reynolds C.R., Kamphaus R.W. (1992). Behavior Assessment System for Children (BASC).

[73] Pentz M.A., Trebow E.A., Hansen W.B., MacKinnon D.P., Dwyer J.H., Johnson C.A., Flay B.R., Daniels S., Cormack C. (1990). Effects of program implementation on adolescent drug use behavior: The Midwestern Prevention Project (MPP). Eval. Rev..

[74] Shillington A., Clapp J. (2000). Self-Report stability of adolescent substance use: Are there differences for gender, ethnicity and age?. Drug Alcohol Depend..

[75] Fite P.J., Colder C.R., Lochman J.E., Wells K.C. (2007). Pathways from proactive and reactive aggression to substance use. Psychol. Addict. Behav..

[76] Pentz M.A., Johnson C.A., Dwyer J.H., Mackinnon D.M., Hansen W.B., Flay B. (1989). A Comprehensive Community Approach to Adolescent Drug Abuse Prevention: Effects on Cardiovascular Disease Risk Behaviors. Ann. Med..

[77] Boxmeyer C., Powell N.P., Lochman J.E., Dishion T.J., Wojnaroski M., Winter C. (2015). Cognitive-Behavioral Group Coding System.

[78] Raudenbush S.W., Bryk A.S. (2002). Hierarchical Linear Models: Applications and Data Analysis Methods.

[79] Johnston L.D., Miech R.A., O’Malley P.M., Bachman J.G., Schulenberg J.E., Patrick M.E. (2019). Monitoring the Future Monitoring Survey Results on Drug Use, 1975–2018: 2018 Overview, Key Findings on Adolescent Drug Use.

[80] Diamond A. (2013). Executive Functions. Annu. Rev. Psychol..

[81] Pandina R.J., Johnson V.L., White H.R. (2009). Peer influences on substance use during adolescence and emerging adulthood. Handbook of Drug Use Etiology.

[82] Fite P.J., Colder C.R., Lochman J.E., Wells K.C. (2008). The Relation between Childhood Proactive and Reactive Aggression and Substance Use Initiation. J. Abnorm. Child Psychol..

[83] Levy S., Sherritt L., Harris S.K., Gates E.C., Holder D.W., Kulig J.W., Knight J.R. (2004). Test-Retest Reliability of Adolescents’ Self-Report of Substance Use. Alcohol. Clin. Exp. Res..

[84] Chapman C.L., Baker E.L., Porter G., Thayer S.D., Burlingame G.M. (2010). Rating group therapist interventions: The validation of the Group Psychotherapy Intervention Rating Scale. Group Dyn. Theory Res. Pract..

